# Sirtuin 4 Knockout Aggravates Sepsis‐Induced Acute Liver Injury by Enhancing Mitochondrial Fission and Mitophagy in Hepatocytes

**DOI:** 10.1155/mi/7600668

**Published:** 2026-01-08

**Authors:** Na Li, Dan Ma, Suxin Luo, An He, Shuting Chang

**Affiliations:** ^1^ Department of Cardiovascular Medicine, Cardiovascular Research Center, The First Affiliated Hospital of Chongqing Medical University, No.1 Youyi Road, Yuzhong District, Chongqing, 400016, China, cqmu.edu.cn

**Keywords:** DRP1, mitophagy, Parkin, sepsis-induced liver injury, sirtuin 4

## Abstract

**Background:**

Sepsis leads to multiorgan damage, with the liver being the main target. Sirtuin 4 (Sirt4) plays a regulatory role in mitochondrial function and metabolism, but its mechanism in liver injury caused by sepsis remains unclear.

**Methods:**

The mouse model of liver injury caused by sepsis was established by cecal ligation and puncture (CLP) surgery. The degree of liver injury in wild‐type (WT) and Sirt4 gene total knockout (Sirt4‐KO) mice was compared by serum AST, alanine aminotransferase (ALT), and histological analysis. The expression of mitophagy and mitochondrial dynamic indicators was detected by biochemical experiments.

**Results:**

Liver injury in Sirt4‐KO mice was more severe than that in WT mice after CLP, manifested as significant upregulation of mitophagy and mitochondrial dynamics imbalance. Mechanistically, Sirt4 deficiency increases mitochondrial fission and mitophagy, thereby leading to cellular damage.

**Conclusions:**

Sirt4 knockout (KO) aggravates liver injury in sepsis through increasing mitochondrial fission and mitophagy, which indicates a promising direction for future clinical treatment.

## 1. Introduction

Sepsis, a life‐threatening organ dysfunction caused by host response dysregulation resulting from infection [1, 2], is one of the leading causes of death among patients in intensive care units worldwide [3]. It begins with an excessive systemic inflammatory response caused by pathogen invasion (i.e., “cytokine storm”) [4] and then progresses to immunosuppression, metabolic disorders, and persistent multiple organ dysfunction syndrome (MODS) [5], with a persistently high mortality rate. In MODS, hepatic injury occupies a core position [6]. As the metabolic and immune center of the human body, the liver is highly vulnerable to damage in sepsis. Its functional disorder will further exacerbate the deterioration of the overall condition, creating a vicious cycle. Sepsis‐induced acute liver injury (SALI) severely disrupts the stability of metabolic homeostasis and weakens its ability to clear pathogens, thus forming a vicious cycle, greatly aggravating the overall condition, and indicating a poor clinical prognosis [7]. Therefore, clarifying the molecular mechanism of liver injury in sepsis is crucial for developing new therapeutic strategies.

Mitochondria, as the energy factory of cells and the integration center of apoptotic and inflammatory signals, play a dual role in functional homeostasis in sepsis [8, 9]. Moderate mitophagy is a protective mechanism for eliminating damaged mitochondria and maintaining cellular health. However, excessive or uncontrolled mitochondrial autophagy leads to cellular energy depletion and death [10]. The balance of mitochondrial dynamics (fusion and division) is the foundation for maintaining its function, and excessive division is often associated with cell damage [11]. Sirtuin 4 (Sirt4) is an NAD+–dependent deacetylase located in mitochondria and has been found to play a key role in energy metabolism and stress response in recent years [12]. However, the role of Sirt4 in sepsis, a major stress model, especially its function in the liver, a key organ, has been poorly studied. Whether it participates in liver injury in sepsis by regulating mitochondrial quality control remains unknown. Based on the mitochondrial localization of Sirt4 and its emerging role in the stress response [13], we propose a scientific hypothesis that Sirt4 may play an important protective role in liver injury caused by sepsis by regulating mitochondrial autophagy and kinetic balance.

In this study, a sepsis mouse model was established through cecal ligation and puncture (CLP) surgery [14], and the liver injury phenotypes of wild‐type (WT; C57) mice and Sirt4 gene knockout (Sirt4^-/-^ KO) mice were compared. The results showed that the deficiency of Sirt4 significantly aggravated liver dysfunction and elevated levels of serum inflammatory factors induced by CLP. At the mechanistic level, this study for the first time reveals that the absence of Sirt4 induces mitochondrial dysfunction and hepatocyte damage by intensifying mitochondrial autophagic flux and disrupting mitochondrial dynamic balance (tending towards excessive division). This discovery establishes a brand‐new protective role of Sirt4 in liver injury caused by sepsis, providing a new perspective for understanding the mechanism of organ damage in sepsis and positioning Sirt4 as a potential therapeutic intervention target.

## 2. Methods

### 2.1. Animals and CLP Model

Male C57BL/6J mice, aged 6–8 weeks and weighing between 20 and 25 g were purchased from GemPharmatech in Chengdu, China. Global Sirt4 KO mice were generated, backcrossed onto the C57BL/6J background, and were viable. Mice were housed with a 12 h day–night circadian cycle in a specific pathogen‐free (SPF) barrier system and allowed sterilized water and food ad libitum. After 7‐day adaptive feeding, mice were randomly categorized into the SHAM, CLP, KO SHAM, KO CLP, OE SHAM, and OE CLP groups (*n* = 10). To achieve Sirt4 overexpression (Sirt4‐OE) in vivo, the lentivirus was used. A liver‐targeted AAV8 system containing Sirt4 (provided by OBiO Technology Co., Ltd., Shanghai, China) was delivered into the mice via the tail vein at a dose of 5 × 10^11^ vg (200 μL per mouse) [15]. A sepsis model was induced by the CLP method as previously described [14]. A 1.5 cm incision was made along the length of the lower quadrant of the abdomen of the mice, followed by isolation of the cecum. Finally, ligating the distal three‐quarters of the cecum with 4–0 silk sutures and making two punctures in the ligated cecum with a 22‐gauge needle to induce the sublethal sepsis in male mice. The Sham group underwent the same surgical exposure without CLP. During the observation period following successful modeling, we assessed whether the mice exhibited intolerable pain or significant declines in mobility, which were defined as humane endpoints. At the end of the experiment, all mice were euthanized by being exposed to overdose of isoflurane (≥5%). After the animal stops breathing, exposure must continue for at least 1 min to ensure brain death. All animal handling procedures, including the euthanasia protocol, followed the National Institutes of Health (NIH) guidelines and were approved by the Animal Ethics Committee of Chongqing Medical University (animal ethics approval number: IACUC‐CQMU‐2023−0065). Animal welfare and experimental protocols were conducted in accordance with the ARRIVE guidelines and strictly adhered to the Guide for the Care and Use of Laboratory Animals of the United States.

### 2.2. Enzyme‐Linked Immunosorbent Assay (ELISA)

ELISA kit for detecting Interleukin‐6 (IL‐6) (GEM0001–48 T, ServiceBio, China) was conducted according to manufacturers’ instructions. The OD value was measured at 450 nm.

### 2.3. Biochemical Measurements

Liver functions were analyzed by serum levels of aspartate aminotransferase (AST; C009‐2‐1, Nanjing Jiancheng, China) and alanine aminotransferase (alanine aminotransferase [ALT]; C010‐2‐1, Nanjing Jiancheng, China). The experiments were performed following the manufacturer’s protocols.

### 2.4. Histological Analysis

Liver tissues were fixed in 4% paraformaldehyde and embedded in paraffin; hepatic sections (4‐μm thick) were cut and then stained with an H&E staining kit (C0105M, Beyotime, China) or TUNEL staining kit (KTA2010, Abbkine Scientific, China) to evaluate the histological score and apoptosis, respectively. Immunohistochemistry was performed on the sections with anti‐Sirt4 (1:100, 862208, ZENBIO), anti‐TNF‐α (1:100, 346654, ZENBIO), or anti‐Parkin (1:100, R381626, ZENBIO) antibodies.

### 2.5. Transmission Electron Microscopy (TEM)

Hepatic tissues were cut into 1 mm^3^ pieces and were fixed in 2.5% glutaraldehyde for 2 h at 4 °C. The pieces were postfixed in 1% osmium tetroxide in phosphate buffer (PB) for less than 2 h. After washing three times with PB, the samples were dehydrated, immersed, and embedded overnight at room temperature. The liver sections (50–70 nm) were cut with an ultramicrotome (Leica EM UC7) and stained with uranyl acetate and lead citrate. Finally, the sections were photographed by an HT7700 transmission electron microscope (HITACHI, Tokyo, Japan) at 120 kV. The images of mitochondrial morphology were presented at different magnifications.

### 2.6. Cell Culture and Transfection

The AML12 cell line (TCM‐C709, Hycyte, China) was cultivated at 37 °C with 5% CO_2_ in AML12‐Luc cell‐specific culture medium (TCM‐G709L, Hycyte, China) [16]. SiRNA was transfected into each dish according to the operating instructions. Sirt4 small interfering RNA and control siRNA were purchased from GenePharma (Shanghai, China) and transfected with Lipomaster‐2000 from Vazyme (Nanjing, China) following the manufacturer’s instructions. The siRNA‐Sirt4 sequence was sense 5′ → 3′: CCAAGAAACUCCUCGUGAUTT and antisense5′→3′: AUCACGAGGAGUUUCUUGGTT. The negative control (NC) sequence of siRNA was sense: UUCUCCGAACGUGUCACGUTT and antisense: ACGUGACACGUUCGGAGAATT. After being transfected with siRNA for 24 h, AML12 was treated by Mdivi‐1(50 μM, MCE, HY‐15886) for 2 h and subsequently stimulated with LPS for 24 h. The overexpression of Sirt4 was conducted by transfecting with the plasmid pLV3‐CMV‐Sirt4(mouse)‐Puro (P83383, MAIOLING, China) in AML12. The efficiency of the above operation in cells was determined by western blotting.

### 2.7. JC‐1 Staining

The mitochondrial membrane potential (MMP) was detected by the JC‐1 staining kit (C2006, Beyotime, China) according to the manufacturer’s protocols. Briefly, the decline of cell membrane potential can be easily detected by the transformation of JC‐1 from red fluorescence to green fluorescence. Meanwhile, the transformation of JC‐1 from red fluorescence to green fluorescence can also be used as a detection index for the early stage of apoptosis [17].

### 2.8. Propidium Iodide (PI)/Hoechst and Immunofluorescent Staining

Cells were inoculated in a 12‐well plate with 2.5 × 10^5^ cells per well and administered the specified compounds. After incubation, the media was removed, and the PI/Hoechst kit (5 μg/mL, C1375S, Beyotime) was used to stain the cells for 30 min as per manufacturer’s instructions. Immunofluorescence was executed with anti‐Parkin antibody (1:100, R381626, ZENBIO) in AML12 cells. Images were obtained using a fluorescence microscope.

### 2.9. Flow Cytometry (FCM)

The cell sample suspensions were washed twice with PBS, stained with the indicated antibodies for 30 min on ice, and subjected to FCM analysis. Annexin V‐FITC/PI apoptosis detection kit (C1062M, Beyotime, China) was used as a probe with annexin V‐labeled FITC.

### 2.10. Western Blotting

Liver tissues and AML12 cells were lysed by RIPA lysis solution (P10013; Beyotime, China) containing protease inhibitors. The protein extracts were then separated via SDS‒PAGE and placed on membranes. The membranes were incubated with primary antibodies: anti‐Sirt4 (1:1000, 69786S, Cell Signaling Technology), anti‐IL‐1β (1:1000, 16806‐1‐AP, Proteintech), anti‐TNF‐α (1:1000, 346654, ZENBIO), anti‐Parkin (1:1000, R381626, ZENBIO), anti‐PINK1 (1:1000, 507131, ZENBIO), anti‐MFF (1:1000, R389288, ZENBIO), anti‐DRP1 (1:1000, 221099, ZENBIO), anti‐OPA1 (1:1000, R382025, ZENBIO), anti‐LC3B (1:1000, R381544, ZENBIO), anti‐MFN1 (1:1000, 13798‐1‐AP, Proteintech, China), anti‐TOM20 (1:5000, 11802‐1‐AP, Proteintech, China), and anti‐β‐actin (1:20000, 66009‐1‐Ig, Proteintech, China) overnight at 4 °C. The appropriate secondary antibodies were incubated at room temperature for 1 h. Chemi Doc Touch Imaging System (Bio‐Rad, Hercules, CA, USA) and ImageJ software (NIH, Bethesda, MD, USA) were used for collecting images and quantification, respectively.

### 2.11. Statistical Analysis

All the quantitative data are presented as the means ± standard errors (SEMs). The findings were analyzed using Prism 8.2.1 software (GraphPad, San Diego, CA, USA). Two‐tailed Student’s *t*‐tests were used to compare the results from two groups. One‐way analysis of variance was taken to compare the results from several groups with the Tukey test for post hoc analysis. *p* value < 0.05 was regarded as statistical significance.

## 3. Results

### 3.1. Sirt4 Is Downregulated in CLP–Treated Mouse Liver and in LPS–Treated AML12

To determine the expression of Sirt4 in the livers of CLP–treated mice, we established a sepsis‐associated acute liver injury (ALI) model by CLP. Immunohistochemical staining indicated that the Sirt4 intensity was downregulated from 42.5% in the SHAM group to 20.2% in the CLP group (Figure 1A,B; *n* = 6, *p*  < 0.0001). Immunoblotting demonstrated that Sirt4 was decreased about 50% after CLP relative to the SHAM group (Figure 1C,D; *n* = 6, *p*  < 0.0001). AML12 was treated by different concentrations of LPS to mimic the sepsis‐induced ALI in vitro (Figure 1E). As a result, AML12 was stimulated with LPS at a concentration of 8 μg/mL for 24 h led to a significant depression of Sirt4 (Figure 1F; *n* = 6, *p*  < 0.0001).

Figure 1Sirt4 is downregulated in the liver of CLP–treated mice in vivo and LPS–treated AML12 in vitro. (A) Immunohistochemical staining of liver sections with Sirt4 antibody in the C57 mouse treated by SHAM or CLP. Scale bar = 100 μm. (B) Quantification of the Sirt4^+^ area (brown, *n* = 6). (C) Western blotting of Sirt4 protein level in the liver of mice at each group. (D) Quantification of the relative protein level in the Sham and CLP group (*n* = 6).  ^∗∗∗∗^ *p* < 0.0001 vs. the SHAM group. (E–F) Immunoblotting analysis of Sirt4 in the AML12 cell line at different concentrations and quantification of relative protein level (*n* = 6). Data are shown as mean ± SEM, and *n* represents the number of samples in each group.  ^∗∗∗∗^ *p* < 0.0001 vs. the Ctrl group.

**Figure:**
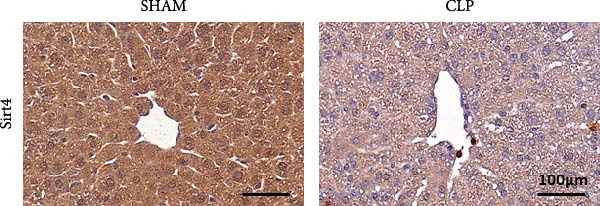
(A)

**Figure:**
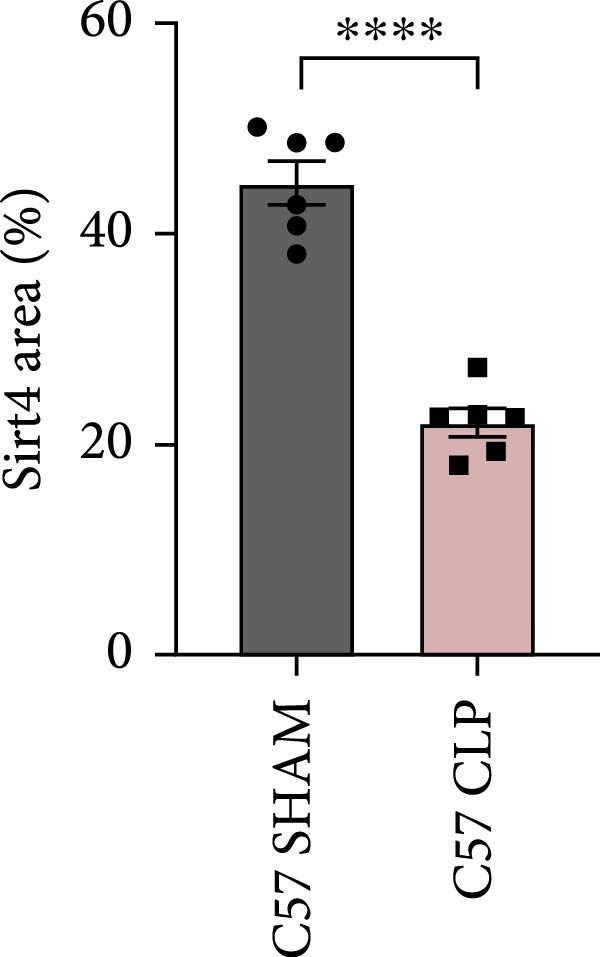
(B)

**Figure:**
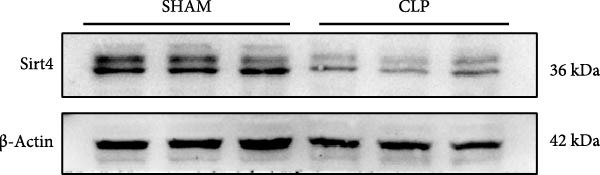
(C)

**Figure:**
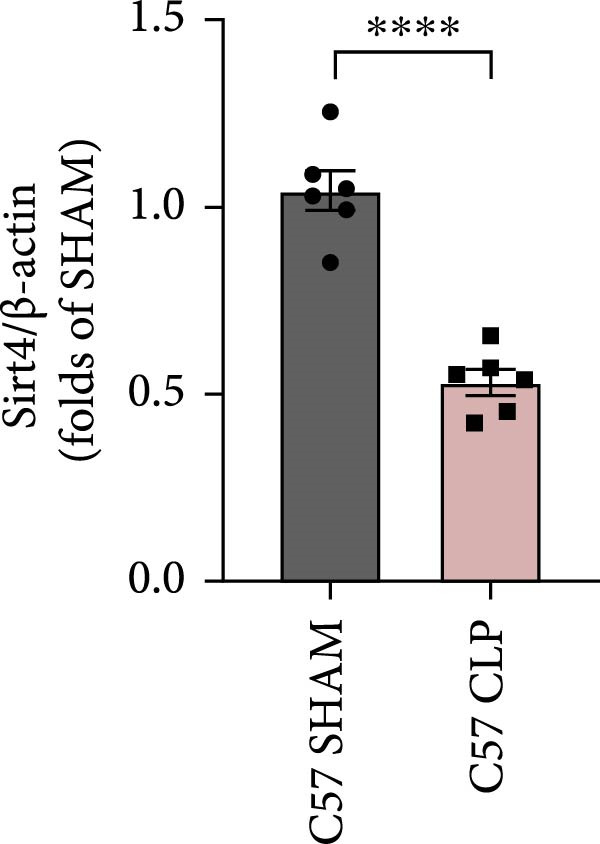
(D)

**Figure:**
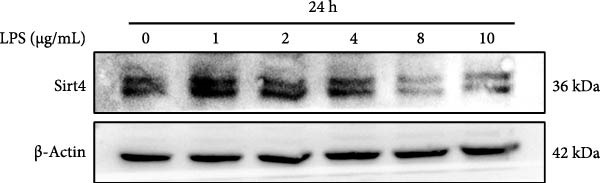
(E)

**Figure:**
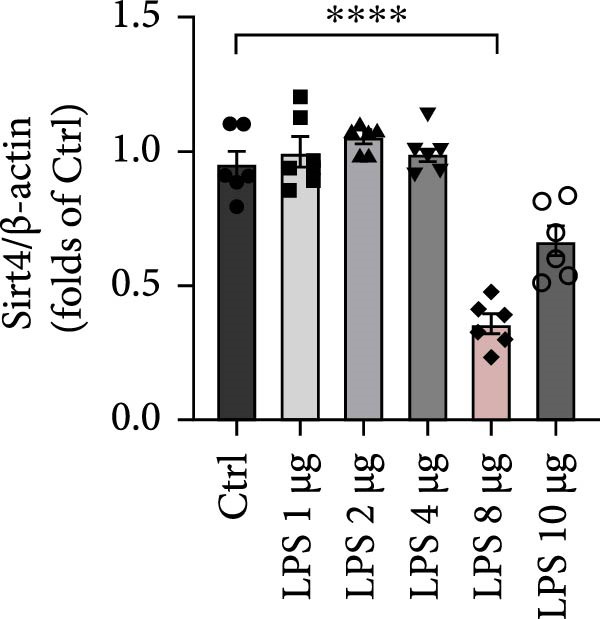
(F)

### 3.2. KO of Sirt4 Aggravates CLP–Induced ALI In Vivo

To figure out the role of Sirt4 in sepsis‐induced liver injury, we generated global Sirt4‐KO mice and subjected them to CLP (Figure 2A). Liver tissues and serum were harvested at 24 h after CLP. Marked inflammation and liver dysfunction were observed after CLP (Figure 2B), as indicated by elevated IL‐6 (*n* = 6, *p*  < 0.01), AST (*n* = 6, *p*  < 0.0001), and ALT (*n* = 6, *p*  < 0.001) levels in mouse serum compared to the SHAM group. Furthermore, this effect was significantly evoked in Sirt4‐KO mice related to C57 mice after CLP surgery (*p* < 0.01, *n* = 6). H&E analysis revealed Sirt4‐KO CLP mice had more severe histological score, which indicated by necrosis, thrombus formation, and infiltrated inflammatory cells, than that in the CLP mice (Figure 2C,D; *n* = 6, *p*  < 0.0001). One of the inflammatory markers, TNF‐α, was much more secreted in the CLP mouse liver than that in the SHAM group (Figure 2E,F; *n* = 6, *p*  < 0.0001), which was further exacerbated in Sirt4‐KO mice (*n* = 6, *p*  < 0.0001). Moreover, TUNEL staining revealed that hepatic apoptosis was substantially elevated in Sirt4‐KO mice than that in the WT mice after CLP (Figure 2G,H; *n* = 6, *p*  < 0.01).

Figure 2Knockout of Sirt4 aggravates CLP–induced liver dysfunction, inflammation, and apoptosis. (A) Schematic diagram of WT or Sirt4‐knockout (KO) mice subjected to CLP for 24 h. (B) The levels of serum IL‐6, AST, and ALT in each group (*n* = 6). (C–D) H&E staining of liver sections and quantification of histological score (*n* = 6). Scale bar = 50 μm. (E–F) Immunohistochemical staining of liver sections using anti‐TNF‐α antibody (brown area) and quantification of TNF‐α+ area (*n* = 6). Scale bar = 50 μm. (G–H) TUNEL (green) and DAPI (blue) staining of liver sections and quantification of TUNEL + area (*n* = 6). Data are shown as mean ± SEM, and *n* represents the number of samples in each group. Scale bar = 50 μm.  ^∗^ *p* < 0.05,  ^∗∗^ *p* < 0.01, and  ^∗∗∗∗^ *p* < 0.0001 vs. the SHAM group; ^**##**^ *p* < 0.01 and ^**####**^ *p* < 0.0001 vs. the CLP group.

**Figure:**
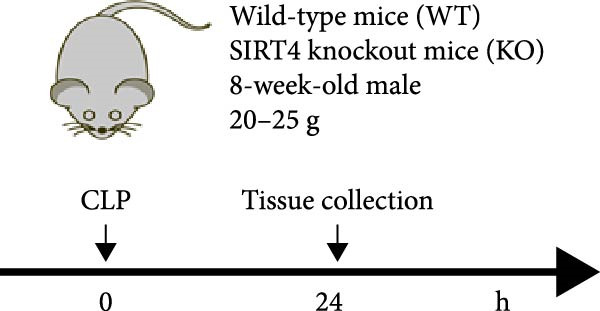
(A)

**Figure:**
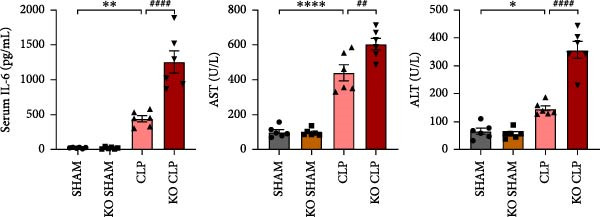
(B)

**Figure:**
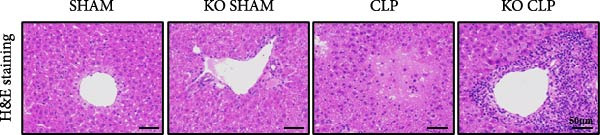
(C)

**Figure:**
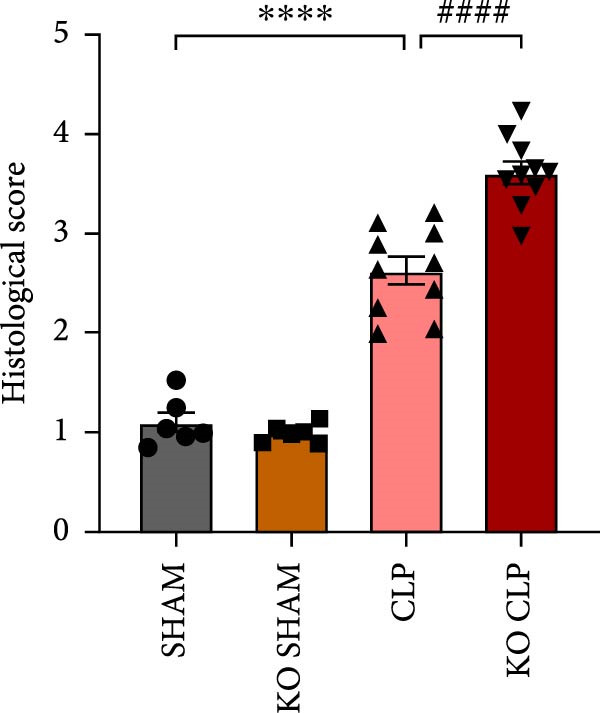
(D)

**Figure:**
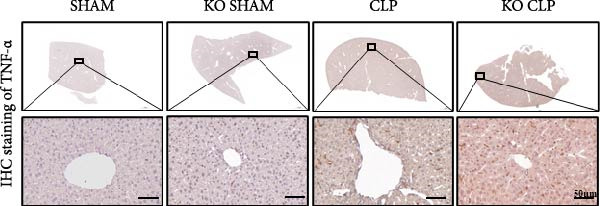
(E)

**Figure:**
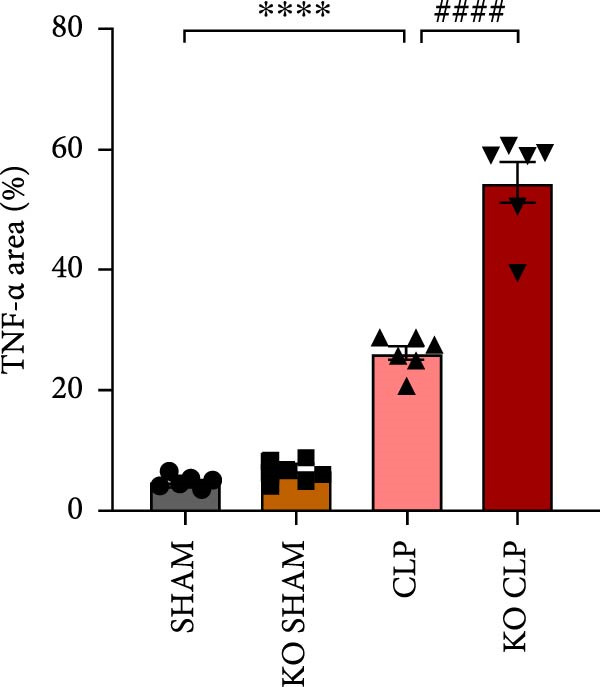
(F)

**Figure:**
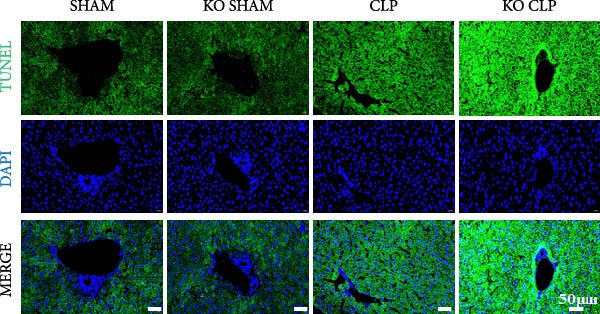
(G)

**Figure:**
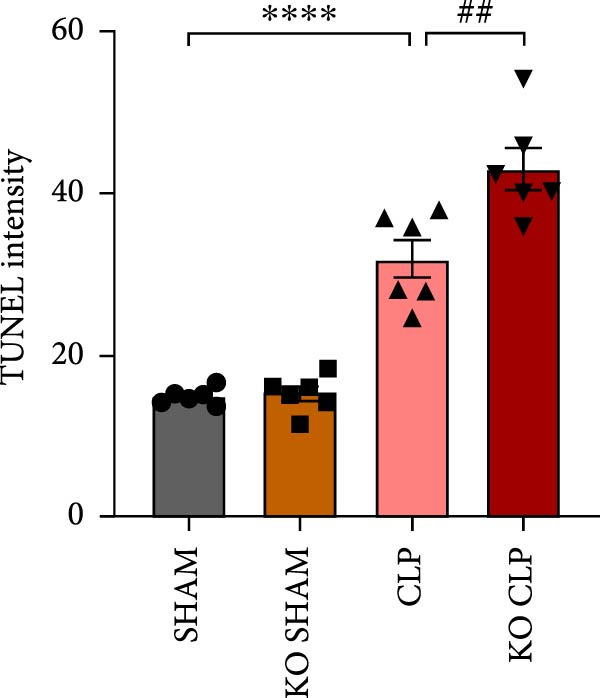
(H)

### 3.3. KO of Sirt4 Exacerbates Mitochondrial Injury by Promoting Imbalanced Mitochondrial Dynamics and Mitophagy in Hepatocytes

Mitochondrial dysfunction triggers apoptosis of hepatocytes [18]. Therefore, we investigated whether Sirt4 KO affects CLP–induced hepatic inflammation and apoptosis via mitochondrial damage. As shown in Figure 3A, sharply decreased mitochondrial cristae and increased fission were observed in Sirt4‐KO mice liver relative to the C57 group after CLP treatment (Figure 3B; *n* = 6, *p*  < 0.05). Mitophagy closely relates to mitochondrial damage [19], so that we detected the expression level of mitophagy in mouse liver. Immunohistochemical staining of mitophagy‐associated marker, Parkin, was upregulated in the Sirt4‐KO CLP mice liver relative to the CLP group (Figure 3C‐D; *n* = 6, *p*  < 0.0001). Accordingly, immunoblotting assays showed the expression of Parkin and LC3B in the Sirt4‐KO mice liver was significantly increased compared to the CLP group (Figure 3E; *n* = 6, *p*  < 0.0001). In addition, mitophagy was mainly affected by mitochondrial dynamics, including the mitochondrial fission‐associated and the fusion‐associated proteins [20]. Compared with the CLP group, the mitochondrial fission‐related markers (DRP1 and MFF) were upregulated, whereas the mitochondrial fusion‐related marker (MFN1) was downregulated in the CLP KO group, respectively (Figure 3F; *n* = 6, *p*  < 0.0001). Consistently, immunoblotting analysis showed the expression of Parkin and LC3B was upregulated by Sirt4 knockdown compared with the NC group after LPS treatment in vitro (Figure 3G; *n* = 6, *p*  < 0.0001). Figure 3F shows the expression of MFF and DRP1 was upregulated, while the expression of MFN1 was downregulated by Sirt4 knockdown compared with the NC group after LPS treatment (*n* = 6, *p*  < 0.01).

Figure 3Sirt4 knockout exacerbates mitochondrial damage in CLP–induced hepatic injury. (A) Representative TEM images of mitochondria in the CLP–treated C57 or Sirt4‐KO mouse liver. Red circles mark the location of mitochondria chosen in cells. Arrows mark the mitochondrial cristae. Scale bar = 500 nm. (B) Quantification of mitochondrial cristae in different groups (*n* = 6). (C) Immunohistochemical staining of liver sections using anti‐Parkin antibody (brown area). (D) Quantification of the Parkin + area (*n* = 6). Scale bar = 100 μm. (E) Western blotting and statistical analysis of mitophagy proteins in mouse liver (*n* = 6). (F) Western blotting and statistical analysis of mitochondrial dynamic proteins in mouse liver (*n* = 6).  ^∗∗∗∗^ *p* < 0.0001 vs. the SHAM group; ^**#**^ *p* < 0.05 and ^**####**^ *p* < 0.0001 vs. the CLP group. (G) Western blotting and statistical analysis of mitophagy proteins in AML12 (*n* = 6). (H) Western blotting and statistical analysis of mitochondrial dynamic proteins in AML12 (*n* = 6).  ^∗∗∗^ *p* < 0.001 and  ^∗∗∗∗^ *p* < 0.0001 vs. the NC Ctrl group; ^**##**^ *p* < 0.01, ^**###**^ *p* < 0.001, and ^**####**^ *p* < 0.0001 vs. the NC LPS group.

**Figure:**
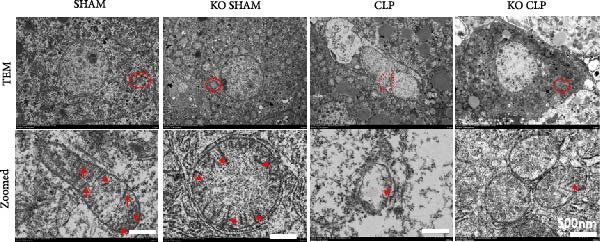
(A)

**Figure:**
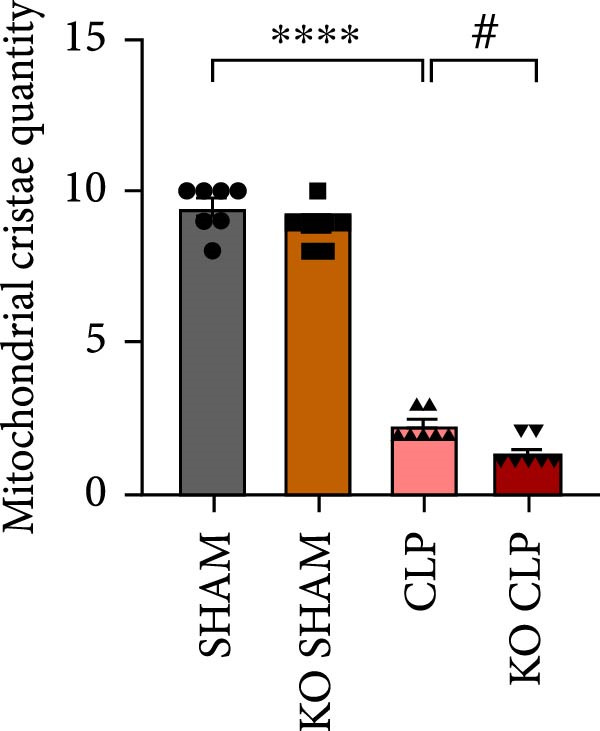
(B)

**Figure:**
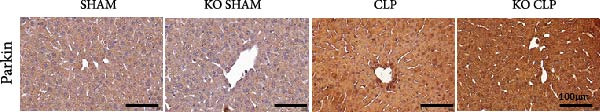
(C)

**Figure:**
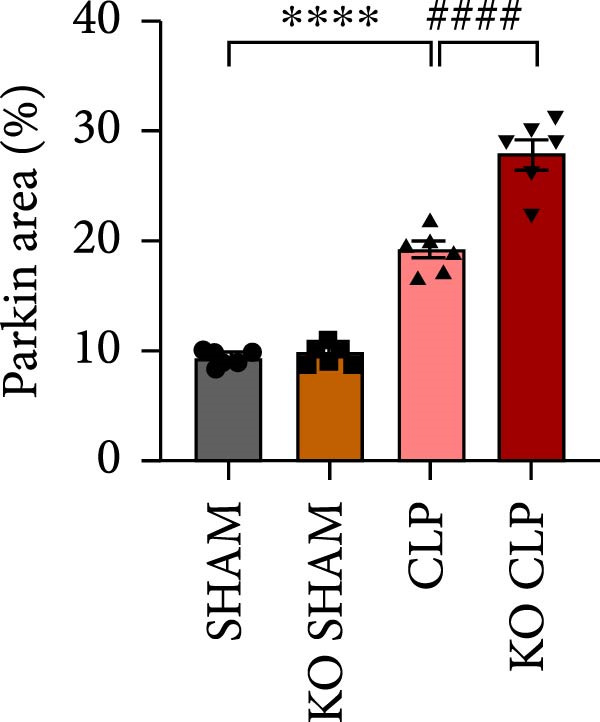
(D)

**Figure:**
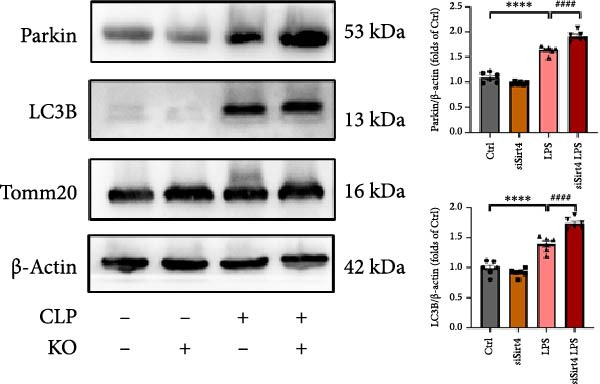
(E)

**Figure:**
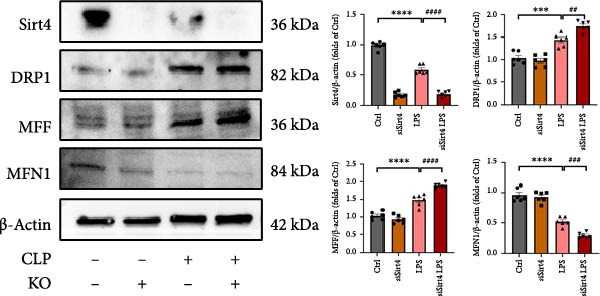
(F)

**Figure:**
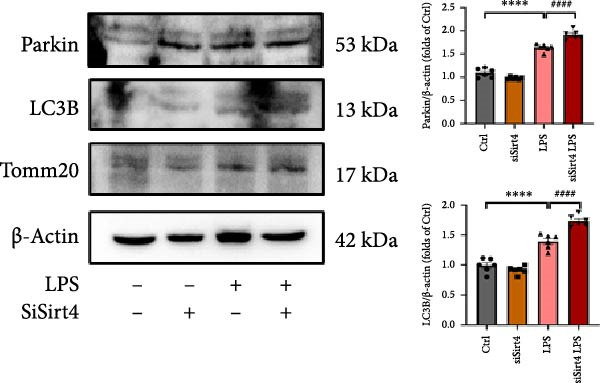
(G)

**Figure:**
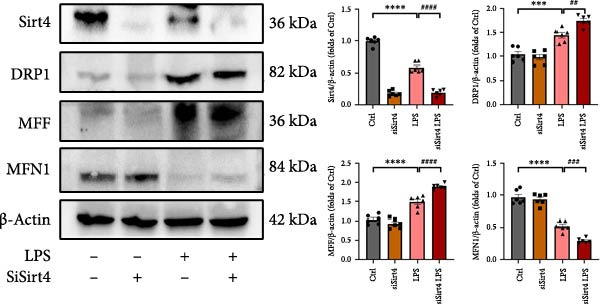
(H)

### 3.4. Sirt4‐OE Alleviates CLP–Induced ALI In Vivo

Sirt4 was overexpressed by lentivirus carrying Sirt4‐OE vectors via tail vein injection prior 1 week to CLP surgery (Figure 4A). Liver tissues and serum were harvested at 24 h after CLP. Marked inflammation and liver dysfunction were observed after CLP (Figure 4B), as indicated by elevated IL‐6 (*n* = 6, *p*  < 0.0001), AST (*n* = 6, *p*  < 0.0001), and ALT (*n* = 6, *p*  < 0.001) levels in mouse serum compared to the SHAM group. However, these effects were obviously attenuated by Sirt4‐OE (*n* = 6, *p*  < 0.001). H&E analysis revealed histological score was reduced in Sirt4‐OE CLP mice liver than that in the CLP mice (Figure 4C,D; *n* = 6, *p*  < 0.0001). Immunohistochemical staining showed TNF‐α was lower secreted in liver of the Sirt4‐OE group than that in the CLP group (Figure 4E,F; *n* = 6, *p*  < 0.001). Moreover, TUNEL staining revealed that hepatic apoptosis was alleviated by Sirt4‐OE than that in the C57 mice after CLP (Figure 4G,H; *n* = 6, *p*  < 0.0001).

Figure 4Overexpression of Sirt4 alleviates CLP–induced liver dysfunction, inflammation, and apoptosis. (A) Schematic diagram of C57 or Sirt4–overexpressed mice subjected to CLP for 24 h. (B) The levels of serum IL‐6, AST, and ALT in each group (*n* = 6). (C–D) H&E staining of liver sections and quantification of histological score (*n* = 6). Scale bar = 50 μm. (E–F) Immunohistochemical staining of liver sections using anti‐TNF‐α antibody (brown area) and quantification of TNF‐α + area (*n* = 6). Scale bar = 50 μm. (G–H) TUNEL (green) and DAPI (blue) staining of liver sections and quantification of TUNEL + area (*n* = 6). Data are shown as mean ± SEM, and *n* represents the number of samples in each group. Scale bar = 50 μm. ^∗∗∗∗^ *p* < 0.0001 vs. the SHAM group; ^###^ *p* < 0.001 and ^####^ *p* < 0.0001 vs. the CLP group.

**Figure:**
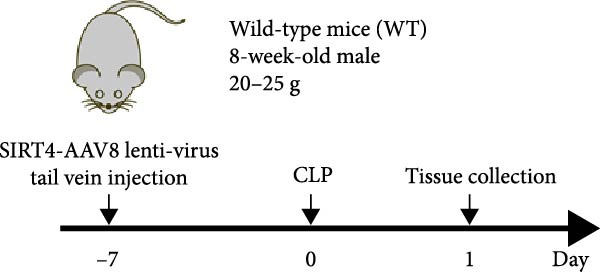
(A)

**Figure:**
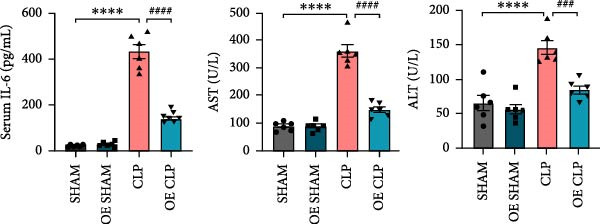
(B)

**Figure:**
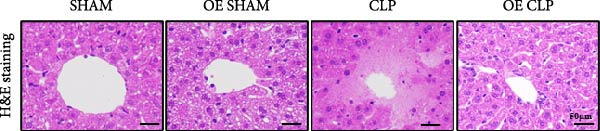
(C)

**Figure:**
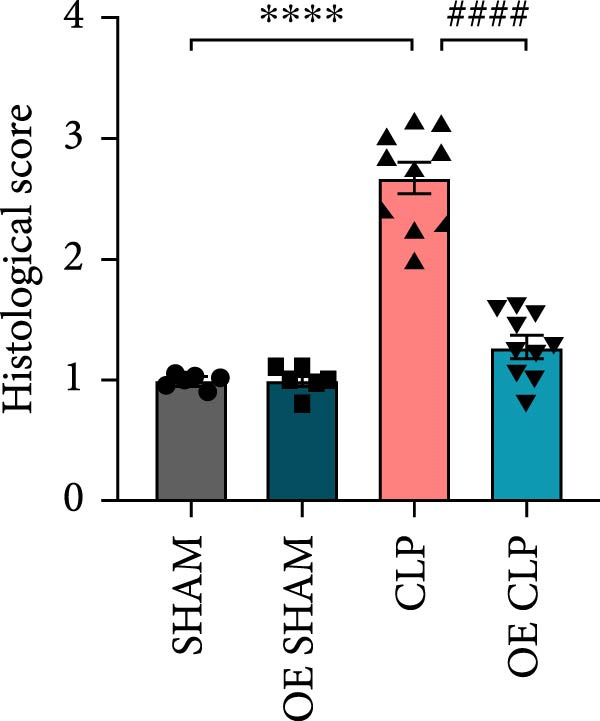
(D)

**Figure:**
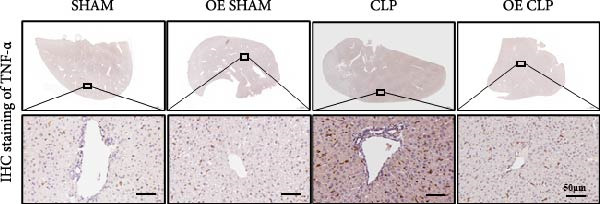
(E)

**Figure:**
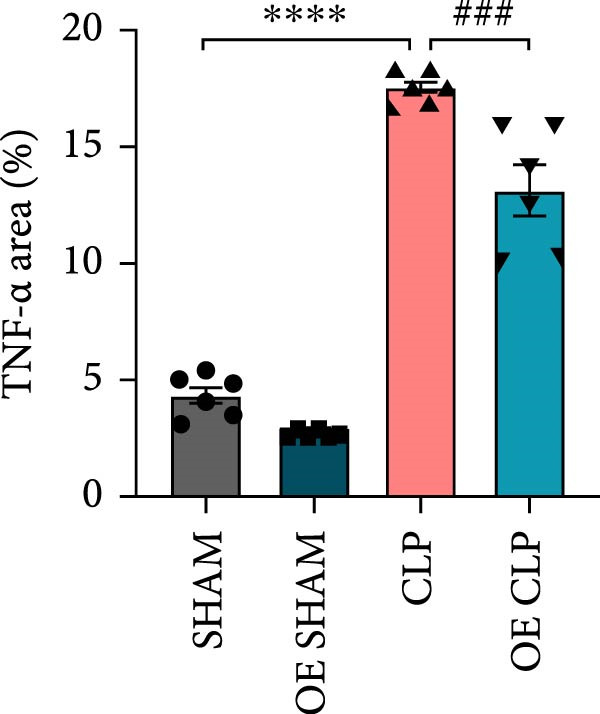
(F)

**Figure:**
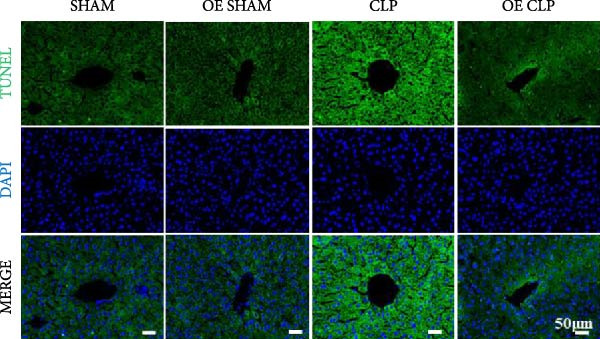
(G)

**Figure:**
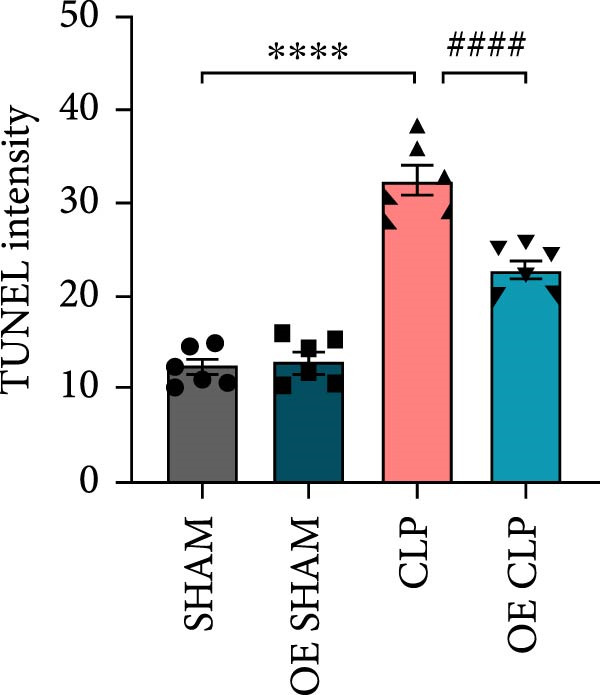
(H)

### 3.5. Overexpression of Sirt4 Attenuates Inflammation and Mitochondrial Injury by Inhibiting Imbalanced Mitochondrial Dynamics and Mitophagy in Hepatocytes

As shown in Figure 5A, mitochondrial cristae were increased in Sirt4‐OE mice liver relative to the C57 group after CLP surgery (Figure 5A,B; *n* = 6, *p*  < 0.05). The expression of Parkin was suppressed by overexpression of Sirt4 in CLP–induced liver injury (Figure 5C,D; *n* = 6, *p*  < 0.0001). Similarly, immunoblotting analysis showed an inhibitory effect of Sirt4‐OE on the expression of mitophagy markers, Parkin and LC3B (Figure 5E; *n* = 6, *p*  < 0.0001). In addition, Sirt4‐OE inhibited the expression of mitochondrial fission factors in mouse liver induced by CLP (Figure 5F; *n* = 6, *p*  < 0.0001) while improving the expression of mitochondrial fusion factor, MFN1 (*n* = 6, *p*  < 0.001). In vitro experiment, we overexpressed Sirt4 in AML12 by transfected with plasmid. The expression of mitophagy and mitochondrial dynamics proteins was detected by immunoblotting (Figure 5G,H). The result showed that Parkin and LC3B were inhibited by Sirt4‐OE in AML12 after LPS stimulation (*n* = 6, *p*  < 0.0001). DRP1 and MFF were depressed, whereas MFN1 was upregulated by Sirt4‐OE in LPS–treated AML12 (*n* = 6, *p*  < 0.0001). Results above suggested a protective role of Sirt4 in SALI through inhibiting mitophagy and regulating mitochondrial dynamics.

Figure 5Sirt4 overexpression improves mitochondrial integrity in CLP–induced hepatic injury. (A) Representative TEM images of mitochondria in the CLP–treated C57 or Sirt4‐OE mouse liver. Red circles mark the location of chosen mitochondria in cells. Arrows mark the mitochondrial cristae. Scale bar = 500 nm. (B) Quantification of mitochondrial cristae in different groups (*n* = 6). (C) Immunohistochemical staining of liver sections using anti‐Parkin antibody (brown area). (D) Quantification of the Parkin + area (*n* = 6). Scale bar = 100 μm. (E) Western blotting and statistical analysis of mitochondrial dynamic proteins in mouse liver (*n* = 6). (F) Western blotting and statistical analysis of mitophagy proteins in mouse liver (*n* = 6).  ^∗∗^ *p* < 0.01 and  ^∗∗∗∗^ *p* < 0.0001 vs. the SHAM group; ^**###**^ *p* < 0.001 and ^**####**^ *p* < 0.0001 vs. the CLP group. (G) Western blotting and statistical analysis of mitochondrial dynamic proteins in AML12 (*n* = 6). (H) Western blotting and statistical analysis of mitophagy proteins in AML12 (*n* = 6).  ^∗^ *p* < 0.05 and  ^∗∗∗∗^ *p* < 0.0001 vs. the NC Ctrl group; ^**####**^ *p* < 0.0001 vs. the NC LPS group.

**Figure:**
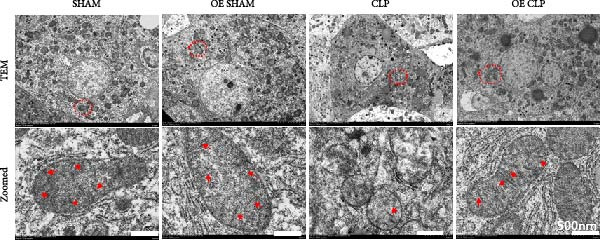
(A)

**Figure:**
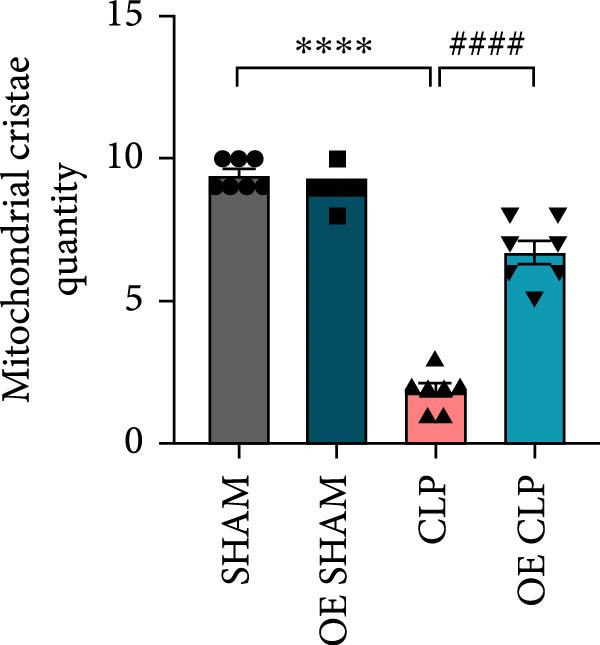
(B)

**Figure:**
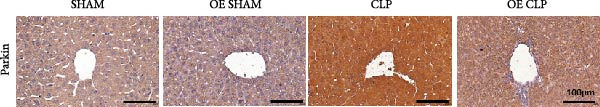
(C)

**Figure:**
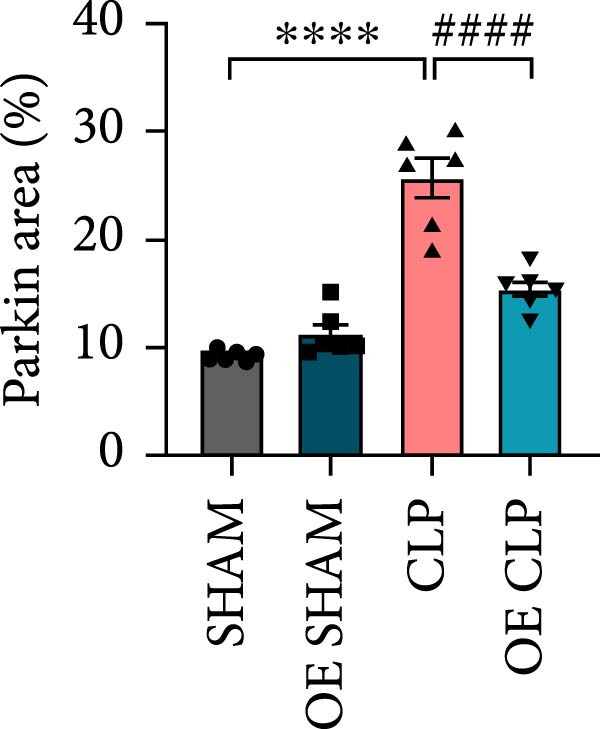
(D)

**Figure:**
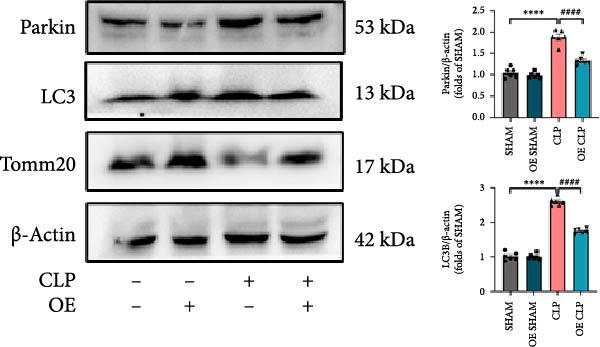
(E)

**Figure:**
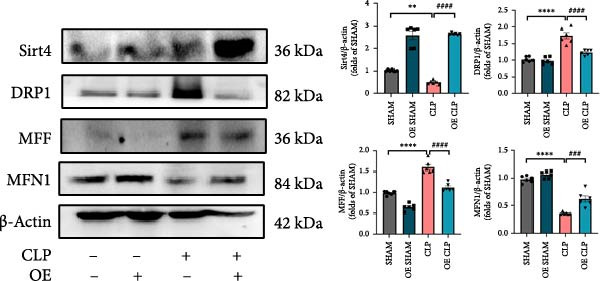
(F)

**Figure:**
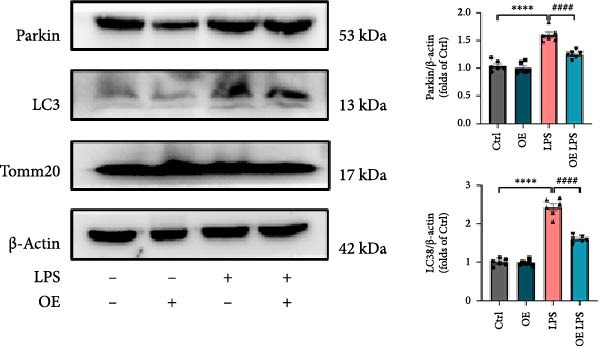
(G)

**Figure:**
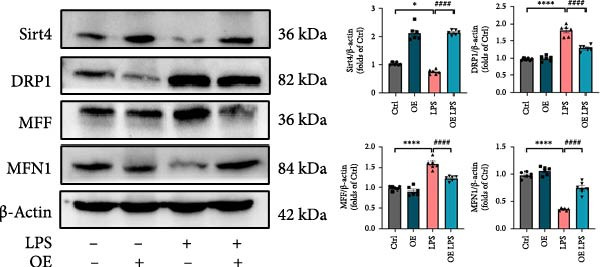
(H)

### 3.6. Mdivi‐1, an Inhibitor of DRP1, Inhibites Cell Damage and Mitochondrial Injury Caused by Sirt4 Deficiency in Hepatocytes

To examine whether Sirt4 mediates mitochondrial function by regulating mitochondrial fission, AML12 was stimulated by Mdivi‐1 after siSirt4 transfection combined with LPS treatment. FCM illustrated that Mdivi‐1 alleviates apoptosis caused by LPS + siSirt4 in AML12 than the group without Mdivi‐1 treatment (Figure 6A,B; *p*  < 0.0001, *n* = 6). JC‐1 ratio showed sharply decreased MMP in the LPS–treated AML12 than the Ctrl group (Figure 6C; *p*  < 0.0001, *n* = 6). MMP was further decreased in the Sirt4 knockdown than the NC group after LPS stimulation. However, Mdivi‐1 improved MMP in AML12 compared with the group without Mdivi‐1 treatment (Figure 6C,D; *p*  < 0.0001, *n* = 6). Furthermore, mitophagy factors including Parkin, PINK1, and LC3B were depressed by Mdivi‐1 in AML12 related to the group without Mdivi‐1 treatment (Figure 6E,F; *p*  < 0.0001, *n* = 6). DRP1 and MFF were inhibited, while MFN1 was improved by Mdivi‐1 in AML12 than the group without Mdivi‐1 treatment (Figure 6G,H;*p* < 0.001, *n* = 6). Immunofluorescent staining of Parkin showed a consistent trend (Figure 6I,J). The immunofluorescent intensity of Parkin was abrogated by Mdivi‐1 versus without Mdivi‐1 group in AML12 (*p* < 0.0001, *n* = 6).

Figure 6An inhibitor of DRP1, Mdivi‐1 suppressed cell damage and mitochondrial injury in AML12 after Sirt4 knockdown and LPS treatment. (A–B) Flow cytometry of apoptosis in LPS–treated AML12 treated with siSirt4 and Mdivi‐1 (*n* = 6). (C–D) Representative images and quantitative analysis of AML12 cells treated with JC‐1 showing aggregates (red) and monomers (green) and the merged fluorescence signals to evaluate the mitochondrial membrane potential (scale bar = 20 μm; *n* = 6). (E–H) Immunoblotting assays and quantitative analysis of mitochondrial dynamic‐associated proteins (E–F) and mitophagy proteins (G–H) in LPS–induced AML12 after siSirt4 and Mdivi‐1 treatment (*n* = 6). (I, J) Immunofluorescent staining and quantitative intensity of Parkin in the LPS–treated AML12 after siSirt4 and Mdivi‐1 treatment (*n* = 6).  ^∗∗∗^ *p* < 0.001 and  ^∗∗∗∗^ *p* < 0.0001 vs. Ctrl + NC group; ^**#**^ *p* < 0.05, ^**##**^ *p* < 0.01, ^**###**^ *p* < 0.001, and ^**####**^ *p* < 0.0001 vs. LPS + NC group; ^&&^ *p* < 0.01 and ^&&&&^ *p* < 0.0001 vs. LPS + siSirt4 group.

**Figure:**
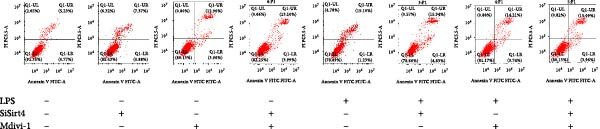
(A)

**Figure:**
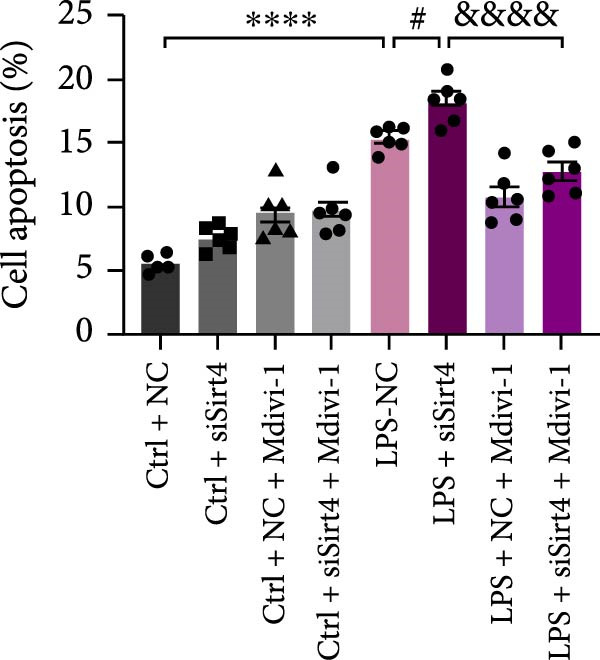
(B)

**Figure:**
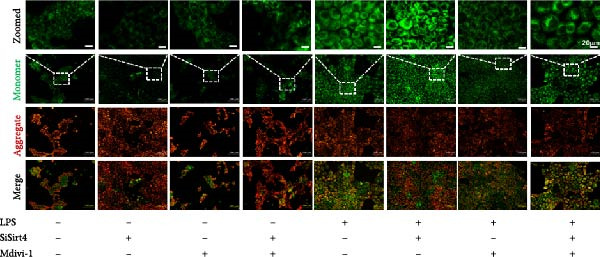
(C)

**Figure:**
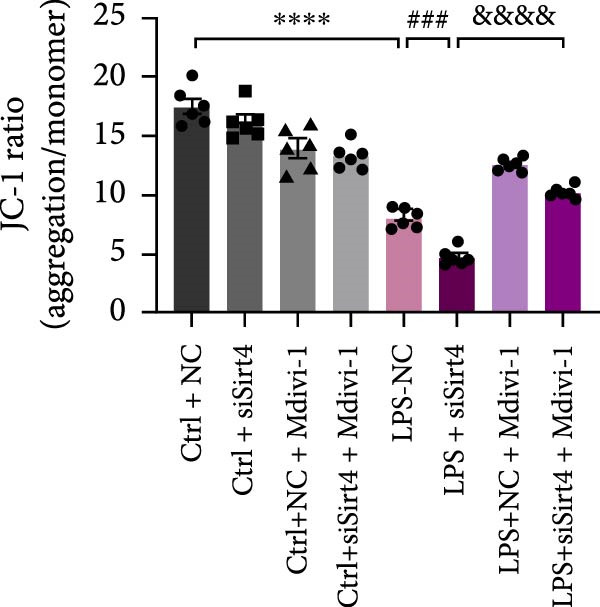
(D)

**Figure:**
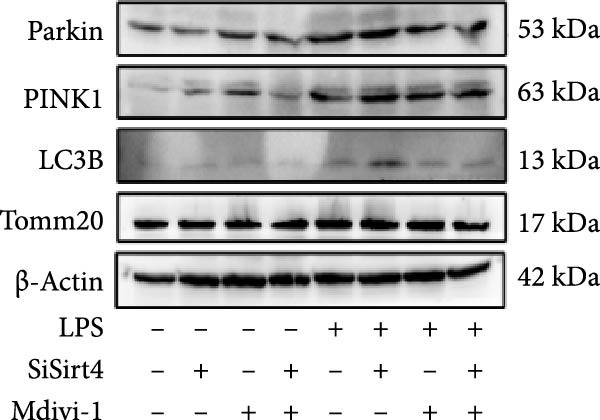
(E)

**Figure:**
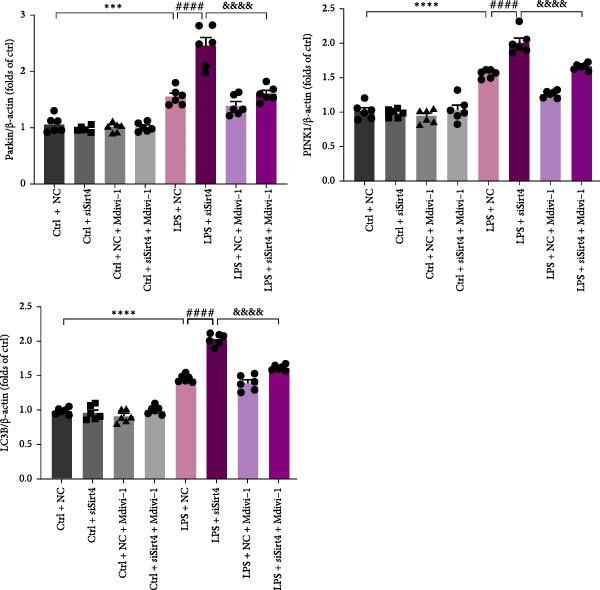
(F)

**Figure:**
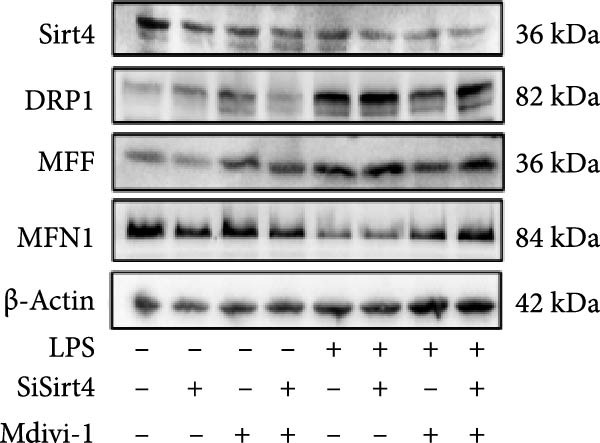
(G)

**Figure:**
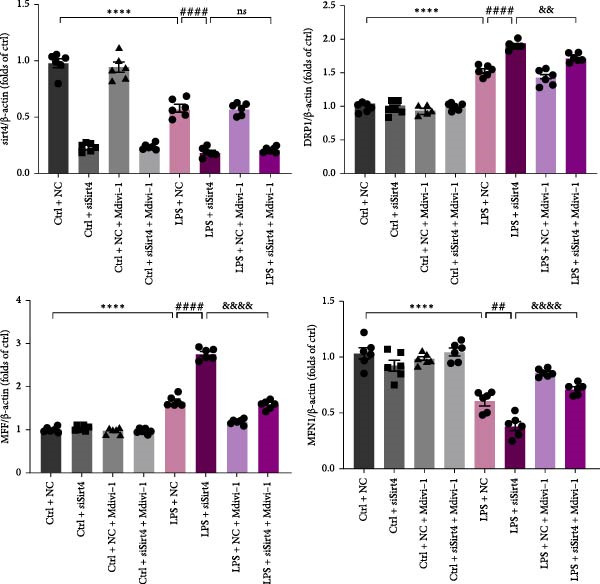
(H)

**Figure:**
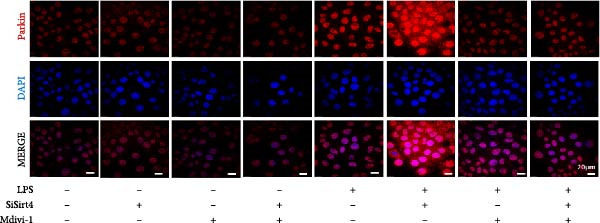
(I)

**Figure:**
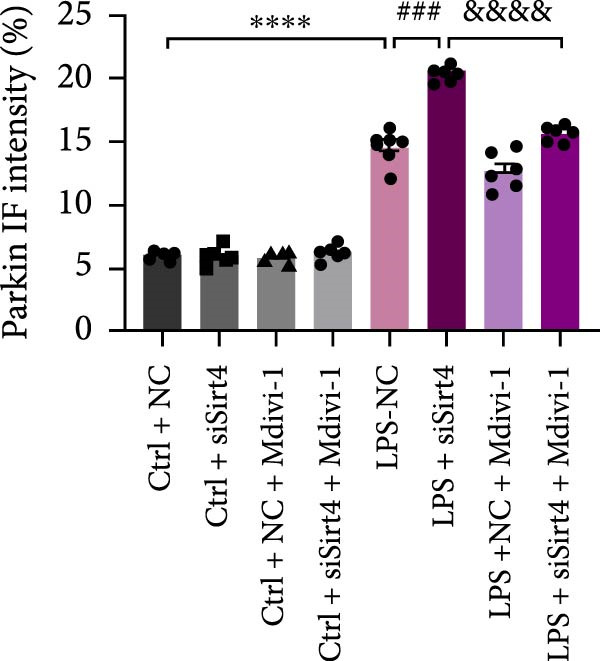
(J)

## 4. Discussion

The main findings of this study are as follows: Firstly, we confirmed that in the CLP–induced sepsis model, the expression of Sirt4 in mouse liver was depressed. Secondly, Sirt4 gene KO mice exhibited more severe liver damage and systemic inflammatory responses than WT mice. The absence of Sirt4 disrupts mitochondrial homeostasis, specifically manifested as overactivated mitochondrial autophagy and imbalanced mitochondrial dynamics (tendency towards division), thereby ultimately leading to more severe hepatocyte damage. Thirdly, Sirt4 regulated mitophagy via suppressing mitochondrial fission induced by sepsis in hepatocyte. This discovery not only establishes the crucial position of Sirt4 in liver protection during sepsis, but more importantly, it directly links Sirt4 to the core pathological process of mitochondrial quality control, providing a promising perspective for clinical treatment of septic liver injury.

Our research is highly consistent with the emerging role of Sirt4 as a guardian of cellular stress [21, 22]. Previous studies on Sirt4 have mostly focused on the metabolic field [23], while this study extends its functional significance to the extreme stress state of sepsis. Sirt4 KO mice exhibited higher levels of serum AST/ALT, more severe liver tissue structure damage, and higher serum concentrations of proinflammatory factor IL‐6 after CLP surgery (Figure 2B), which demonstrates that endogenous Sirt4 is an important molecular barrier against liver injury caused by sepsis. The liver plays a core role in the “immune‐metabolic” crosstalk (interactive dialog) in sepsis [7]. The absence of Sirt4 may simultaneously disrupt the homeostasis of both aspects. On the one hand, damage within hepatocytes directly release damage‐related molecular patterns (DAMPs), further amplifying the systemic inflammatory response [24]. On the other hand, the collapse of liver metabolic function (such as impaired gluconeogenesis and decreased detoxification function) deteriorates the homeostasis and indirectly promote immune disorders [25]. Previous studies mentioned transcription factors involved in the transcriptional regulation of Sirt4 [21, 26]. For instance, STAT2 regulates Sirt4 transcription and activates the mTOR pathway, thereby promoting neuronal apoptosis in Alzheimer’s disease [27]. As for involved posttranslational regulating mechanism, a recent literature reported that glutamine sustains energy metabolism and alleviates liver injury in burn sepsis by promoting the assembly of the mitochondrial HSP60‐HSP10 complex via Sirt4–dependent protein deacetylation [28]. However, the mechanism of the interaction between Sirt4 and mitochondrial homeostatic proteins in septic liver injury has not been reported yet and still requires further exploration.

The most crucial contribution of this study lies in the precise mechanism‐association between Sirt4 and mitochondrial quality control in sepsis. We propose that Sirt4 is a “Calibrator” or “Brake” for mitochondrial autophagy. In WT mice, the normal expression of Sirt4 may modify key proteins in the mitophagy pathway through its ADP–ribosyl transferase activity [29], thereby limiting autophagic flow to a moderate, controllable, and beneficial level, enabling it to effectively clear severely damaged mitochondria while avoiding excessive consumption of healthy mitochondria. Our data indicate that the absence of Sirt4 leads to mitochondrial dynamic imbalance, which is to be manifested as an intensification of mitochondrial fragmentation (Figure 3A), which is usually caused by the excessive increased mitochondrial fission protein DRP1 and decreased fusion factor MFN1 (Figure 3F). Fragmented mitochondria not only have low productivity but are also more unstable, making them more prone to membrane potential collapse and becoming targets of mitophagy (Figure 3C). It is worth noting that there is a close dialog between mitochondrial dynamics and mitophagy: MFF–mediated division is a prerequisite for initiating mitophagy and continuous division promotes extensive mitophagy [30]. Therefore, we observed that excessive mitophagy and mitochondrial fragmentation in Sirt4‐KO mice liver are likely two sides of the same problem, forming a positive feedback loop that intensifies each other and jointly pushes hepatocytes towards death.

The mechanism of Sirt4 regulating both of these processes simultaneously has not been fully revealed. A previous study illustrated that Sirt4 is regulated separately through different substrates: for instance, by modifying DRP1 [31] to inhibit its GTPase activity or mitochondrial recruitment while promoting fusion by modifying OPA1 [32, 33]. Another possibility is that Sirt4 coordinates the remodeling of the entire mitochondrial network by regulating common upstream signals, such as cellular energy status (AMPK/ATP) [34] or oxidative stress levels [35]. Precise identification of the enzymatic activity substrates of Sirt4 in the liver will be the key to future research [36, 37]. In addition, Sirt4 has been revealed to be closely related to different kinds of programed cell death, including apoptosis [38] or ferroptosis [39]. As a key mitochondrial stress sensor, Sirt4 reshapes the function and morphology of mitochondria by integrating metabolic signals (such as glutamine [40] and fatty acid metabolism [41]). Future research should focus on verifying whether Sirt4 directly modifies mitochondrial dynamic‐related proteins and clarifying the specific molecular bridges by which it coordinates these two cell death patterns under specific pathological conditions, such as ischemia‐reperfusion injury [42] and neurodegenerative diseases [43, 44]. The clarification of this mechanism will provide a new theoretical basis and therapeutic strategy for the intervention of related diseases targeting Sirt4.

The results of this study have profound translational medical significance. At present, the treatment of sepsis still mainly focuses on anti‐infection and supportive therapy [45], lacking specific drugs that directly target organ damage. Our research has brought Sirt4 to the center stage as a highly promising novel therapeutic target. Theoretically, by activating the activity of Sirt4 with small molecule drugs, it may be possible to calibrate the uncontrolled mitochondrial autophagy in sepsis, restore it to a protective level, and stabilize the kinetic balance, thereby protecting hepatocytes and even other organ cells from damage and improving the prognosis of patients. However, the road to clinical translation remains long and full of challenges. The primary bottleneck lies in the fact that no highly efficient and specific Sirt4 agonists have been reported so far, which requires major breakthroughs in the fields of medicinal chemistry and chemical biology. Secondly, it is necessary to delve deeply into the role of Sirt4 in different cell types: Is its function consistent in hepatocytes, Kupffer cells, and stellate cells. Will systemic stimulation of Sirt4 have off‐target effects on other organs, such as pancreatic β cells that require Sirt4 to inhibit insulin secretion. Developing a liver‐targeted Sirt4 agonist delivery system might be an ideal solution to this problem.

**Figure 7:**
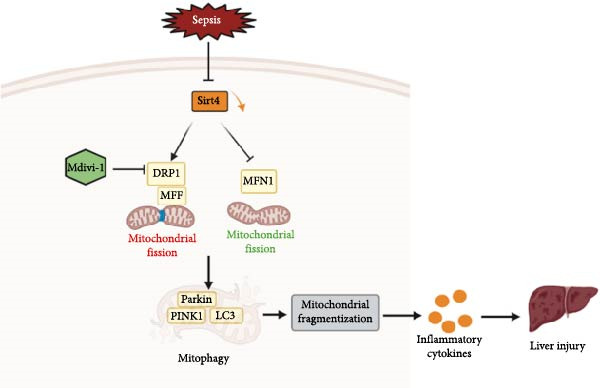
The role of Sirt4 in sepsis‐induced liver injury. Sirt4 was depressed by sepsis in mouse liver, which aggravates unbalanced mitochondrial dynamics including the increased mitochondrial fission and decreased mitochondrial fusion. Mitochondrial dynamics contributes to mitophagy, finally leading to hepatic inflammation and injury.

In conclusion, this study not only established the indispensable role of Sirt4 in ALI caused by sepsis but also revealed a protective mechanism by suppressing mitophagy, which induced by unbalanced mitochondrial dynamics including increased mitochondrial fission and decreased mitochondrial fusion (Figure 7). Our findings delineate a novel Sirt4‐DRP1–mitophagy axis as a critical regulatory mechanism in sepsis‐induced liver injury, suggesting Sirt4 as a potential therapeutic target.

## Ethics Statement

All animal experimental procedures were conducted in accordance with the National Institutes of Health (NIH) guidelines, and ethical approval was obtained from Chongqing Medical University (IACUC‐CQMU‐2023‐0065).

## Disclosure

The author confirms that the work described has not been published previously and is not under consideration for publication elsewhere.

## Conflicts of Interest

The authors declare no conflicts of interest.

## Author Contributions

Na Li performed the experiments and wrote the manuscript and conceived the study. Dan Ma performed the immunofluorescence assays. Suxin Luo and An He designed the experiments and interpreted the data. Shuting Chang wrote the manuscript and supervised the study.

## Funding

This study was supported by the National Natural Science Foundation of China (Grant no. 82300477) and the Doctoral Research and Innovation Project of Chongqing Medical University (Grant number CYYY‐BSYJSKYCXXM202414).

## Data Availability

The data used to support the findings of this study are available from the corresponding author upon request.
